# Prevalence of antibiotic-resistant *E. coli* in broilers challenged with a multi-resistant *E. coli* strain and received ampicillin, an organic acid-based feed additive or a synbiotic preparation

**DOI:** 10.3382/ps/pez004

**Published:** 2019-01-25

**Authors:** Nataliya Roth, Charles Hofacre, Ulrike Zitz, Greg F Mathis, Karl Moder, Barbara Doupovec, Roy Berghouse, Konrad J Domig

**Affiliations:** 1 Department of Food Science and Technology, Institute of Food Science, BOKU—University of Natural Resources and Life Sciences, 1190 Vienna, Austria; 2 Department of Population Health, Poultry Diagnostics and Research Center, University of Georgia, 30602 Athens, Georgia, USA; 3 Institute of Applied Statistics and Computing, BOKU—University of Natural Resources and Life Sciences, 1190 Vienna, Austria; 4 BIOMIN Holding GmbH, 3131 Getzersdorf, Austria; 5 Southern Poultry Research Group, Inc. 30607-3153 Athens, Georgia, USA

**Keywords:** feed additive, poultry, antibiotic resistance, APEC, *E. coli* challenge

## Abstract

The aim of this study was to evaluate the effect of ampicillin, an organic acid-based feed additive and a synbiotic preparation on the prevalence of antibiotic-resistant *E. coli* in the ceca of broilers. A total of 2000 broiler chickens (Ross 708) were randomly assigned to 5 groups with 8 replicates. The negative control group was the only group that was not subjected to avian pathogenic *E. coli* challenge, while all the other 4 groups received a multi-resistant *E. coli* strain that was resistant to ampicillin, cephalexin, and nalidixic acid as an oral challenge. The second group served as a challenge control, and the third group received the antibiotic ampicillin via water for 5 d. The fourth group received a feed additive based on organic acids and cinnamaldehyde, and the fifth group received a synbiotic preparation via feed and water. On day 17 and 38 of the trial, cecal samples from 3 birds from each of the 40 pens were obtained, and the *E. coli* counts and abundances of antibiotic-resistant *E. coli* were determined.

Oral challenge with an avian pathogenic *E. coli* strain did not influence the performance, and there was no significant difference in growth performance between groups. The total *E. coli* count was lower (*P* < 0.05) in the group supplemented with the synbiotic than in the challenge control group on day 38 of the trial. Administration of an antibiotic for 5 d led to a significant increase in the abundance of *E. coli* strains resistant to ampicillin, amoxicillin-clavulanic acid, cefoxitin, and ceftriaxone. There was no increase in the abundance of antibiotic-resistant *E. coli* observed in the groups that received feed supplemented with an organic acid/cinnamaldehyde-based feed additive or a synbiotic. Moreover, the effects of the tested feed additives on the prevalence of resistant *E. coli* are demonstrated by the lower ceftriaxone minimal inhibitory concentration values for this group than for the antibiotic group. Additionally, the synbiotic group exhibited lower ceftriaxone minimal inhibitory concentration values than the antibiotic group.

## INTRODUCTION

Antibiotics have been used for treatment and prevention of disease as well as growth promotion in livestock and poultry production (Allen et al., 2013). The use of antibiotics to treat food-producing animals provides favorable conditions for the spread of antibiotic-resistant (**AR**) bacteria and the corresponding resistance determinants at the farm level (Diarra et al., 2007; Diarrassouba et al., 2007; Miranda et al., 2008; Furtula et al., 2010; da Costa et al., 2011; Burow et al., 2014). The use of antibiotics has potentially increased the prevalence of resistance determinants in animal microbiomes (Pal et al., 2016). The development of resistant pathogens associated with animal diseases has increased, and the growing antibiotic resistance gene pool in commensal bacteria is a cause for concern, and intensive research is required for understanding the prevalence and dynamics of AR bacteria in poultry flocks. *Escherichia coli* is a commensal bacterium in broilers and has a higher prevalence in chicken excreta than some key pathogens (Chinivasagam et al., 2010). *E. coli* may frequently be exposed to selective pressures imposed by antibiotic treatments and may contribute considerably to the spread of antibiotic resistance (Simoneit et al., 2015). Moreover, avian pathogenic *E. coli* (**APEC**) causes various diseases, collectively termed colibacillosis, in chickens, and these diseases are responsible for significant economic loss in the chicken industry (Hammerum and Heuer, 2009; Mohamed et al., 2014). Moreover, poultry products contaminated with APEC are potential sources of foodborne extraintestinal pathogenic *E. coli* infections for humans, posing a threat to human health (Bergeron et al., 2012)

This study evaluated the effect of oral challenge of broilers with a multi-resistant APEC strain and the effects of the antibiotic ampicillin, a feed additive (**FA**) containing organic acids and cinnamaldehyde, and a synbiotic application (**SA**) on the prevalence of AR *E. coli* in the ceca of these broilers. The application in food-producing animals of antibiotics with therapeutically useful analogs has led to public health concerns (Turnidge, 2004; Collignon et al., 2009; da Costa et al., 2011). Ampicillin is an aminopenicillin that is characterized by broad-spectrum antimicrobial activity and is applied in poultry farming for the treatment of bacterial infections (Agunos et al., 2012; Wang et al., 2017). Bacterial resistance to ampicillin has increased significantly since the introduction of this antibiotic in medicine and agriculture in the late 1980s (Everett et al., 1996). The percentage of ampicillin-resistant *E. coli* isolates from broilers in the European Union is approximately 70% (EFSA/ECDC, European Food Safety Authority and European Centre for Desease Prevention and Control, 2016).

Organic acid-based FAs are frequently used in poultry production due to their bactericidal activities, in both feed and the gastrointestinal tract (Ricke, 2003). Organic acids and cinnamaldehyde are known to have antimicrobial activity as well as the ability to promote the growth of chickens (Helander et al., 1998a; Raftari et al., 2009; Wang et al., 2009a; Adil et al., 2010). The effects of non-antibiotic antimicrobial compounds such as organic acids and cinnamaldehyde on resistant *E. coli* are not clear. On the one hand, there is indication that exposure to non-antibiotic antimicrobial agents can induce or select bacterial adaptations that result in decreased susceptibility to one or more antibiotics (Wales and Davies, 2015). On the other hand, the reduction of extended-spectrum cephalosporin-producing *E. coli* has been associated with the use of acidified drinking water in a risk factor study performed in Belgian broiler farms (Persoons et al., 2010). In general, the extent to which antibiotic resistance is associated with the use of chemicals and biological agents—used expressly to control, deter, inhibit, or kill harmful microorganisms—is poorly understood (Food and Agriculture Organization of the United Nations, 2018). This study aims to clarify the effect of FAs based on organic acids as well as synbiotics on resistant *E. coli* in broilers.

Synbiotics may be defined as mixtures of probiotics and prebiotics that beneficially affect the host by improving survival and implantation of live microbial dietary supplements in the gastrointestinal tract via selective stimulation of growth and/or metabolic activation of one or a limited number of health-promoting bacteria, thus improving host welfare (Gibson and Roberfroid, 1995). Probiotics are defined as monocultures or mixed cultures of live microorganisms that beneficially affect the host animal by modulating the gut microbiota in livestock (Fuller, 1989). Prebiotics are defined as non-digestible food ingredients that beneficially affect the host by selectively stimulating the growth and/or activity of one or a limited number of bacteria in the colon (Gibson and Roberfroid, 1995). The application of a synbiotic preparation reduced the *E. coli* and total coliform populations in the intestines of broiler chickens (Dibaji et al., 2014). The antimicrobial activity of organic acid- and cinnamaldehyde-based FAs, as well as the application of a synbiotic preparation, may influence the AR *E. coli* levels in the gastrointestinal tracts of broilers. The present study therefore evaluates the effect of ampicillin and FAs (an organic acid/cinnamaldehyde-based product and a synbiotic preparation) on the prevalence of AR *E. coli* in the gastrointestinal tracts of broilers challenged with a multi-resistant APEC strain.

## MATERIALS AND METHODS

### Animals, Housing and Treatments

The animal experiment was conducted at the facility of the Southern Poultry Research Group (**SPRG**; Athens, GA, USA). A total of 2000 day-of-hatch Ross 708 male broiler chicks were assigned to 5 treatment groups, with 8 replicate blocks, and allocated into groups of 50 birds per pen (pen size was 1.5 meters x 3.0 meters). One empty pen or a 1.5 meter empty space was positioned between the trial pens to reduce cross-contamination. All the animal caretakers wore plastic boots dedicated to each pen and wore gloves when entering the pens.

Treatment groups were assigned to pens using a randomized complete block design. The SPRG completed the randomization and assignment of treatment groups to pens using random permutation tables (Cochran and Cox, 1992). The first group was the negative control (**NC**) and only group without APEC challenge was positioned at a distance of two empty pens from the next group. The second group served as a challenge control (**CC**), and the third group received 100 g of ampicillin trihydrate (**AB**) (= 86.6 g of ampicillin) per 1000 L of water (Ampiciph^®^; bela-pharm GmbH & Co. KG, Germany) from day 11 to day 15 of the trial. The fourth group received a top-dressed FA composed of 20% formic acid, 10% acetic acid, and 5% propionic acid, as well as 2.5% cinnamaldehyde (Biotronic^®^ Top3; BIOMIN Holding GmbH, Austria) at a dose of 2 kg/t of feed during the entire trial period. The fifth SA group received the multistrain synbiotic product PoultryStar^®^ (BIOMIN Holding GmbH, Austria), containing *Enterococcus*, *Pediococcus*, *Bifidobacterium*, and *Lactobacillus* isolated from healthy chicken guts combined with inulin, via feed and water. PoultryStar^®^ me was provided via feed at a concentration of 1 kg/t of feed (2 × 10^8^ CFU/kg of feed), and PoultryStar^®^ sol was provided via drinking water on days 1, 2, 3, 16, 17, and 18 of the trial at a dose of 20 g per 1000 birds per day.

All groups except NC received oral challenge with multi-resistant *E. coli* with resistance to ampicillin, cephalexin, and nalidixic acid. A total of 25 one-day-old chicks per pen were tagged; color-coded for identification; and orally administered (gavage, 0.1 ml into the crop) the APEC strain X-7122, isolated by Dr. Jonn Maurer (Georgia University, USA), at 4.0 × 10^6^ CFU per chick on the first day of the trial. Seeder birds were placed only in treatment groups CC, AB, FA, and SA.

All birds received routine vaccinations and were sprayed with a commercial coccidia vaccine (Advent^®^ Coccidiosis Control; Huvepharma, Bulgaria) at 1 d of age, as per the manufacturer's recommendations.

All birds received a common basal diet without coccidiostats, a starter diet from hatch until day 17 and a grower diet until day 38. Diets were fed as mash throughout the study. The nonmedicated commercial-type broiler starter and grower diets consisted of the feedstuffs commonly used in the United States, which were representative of local formulations and met or exceeded National Research Council (1994) standards. The chicks had free access to feed and water supplied through bell drinkers.

The birds were housed at 0.09 square meters/bird. All birds were subjected to the same rearing, environmental and sanitary conditions. Birds were reared under ambient humidity. Thermostatically controlled gas heaters were the primary heat source. One heat lamp per pen provided supplemental heat during brooding. Birds were provided controlled lighting and ventilation. At placement, each pen contained approximately 4 inches of fresh pine shavings. Litter was not replaced during the course of this study. Each pen contained one tube feeder and one bell drinker (50 birds/feeder and drinker).

### Sampling

To determine total *E. coli* counts and ensure that no multi-resistant *E. coli* strains that were resistant to nalidixic acid, ampicillin, and cephalexin were present in day-of-hatch chicks, swabs of all 20 chick box papers were tested on arrival at the trial facility. Sterile chick box paper was placed on the bottom of the transport box. Chick box papers were aseptically collected at the farm, immediately placed into sterile Whirl Pack bags (Sigma-Aldrich, Germany), and transported on ice to the laboratory for analysis of the presence of AR *E. coli*. On the 17th and 38th days of the trial, 3 chicks per pen were randomly selected and humanely euthanized by using CO_2_. The intestinal tract of each chick was dissected after slaughter, and a total of 240 cecal samples were collected and placed into sterile plastic bags (Fisher Scientific, USA). The samples were labeled, stored on ice, and delivered to the laboratory for *E. coli* analysis.

### Performance Data

Body weight (BW) by pen was calculated as the average of the sum of the weights of 50 birds as determined on days 1, 17, and 38. Average daily weight gain was calculated for day 1 to day 17, day 18 to day 38, and day 1 to day 38 of the trial. Pen-wise feed intake was recorded at day 17 and at the end of the trial on day 38. The average daily feed intake (**ADFI**) was calculated accordingly. The feed conversion rate (**FCR**) was calculated per pen and corrected for mortality.

### Microbiological Analysis

#### E. coli From Chick Papers.

Intestinal samples were kept on ice during transport to the laboratory. Each chick paper was hand swabbed with a premoistened 4 × 4 gauze pad that was then placed into 50 ml of phosphate-buffered saline (**PBS**; Dulbecco's PBS, MP Biomedicals, Solon, Ohio, USA). One milliliter of PBS was spread plated onto MacConkey agar (Becton, Dickinson and Company, Sparks, Maryland, USA) containing 25 μg/ml nalidixic acid, 6.25 μg/ml ampicillin, and 25 μg/ml cephalexin.

#### E. coli Isolation, Identification, and Enumeration.

For all samples, a 1-ml aliquot of PBS was transferred to three adjacent wells in the first row of a 96-well 2-ml-deep block. A 0.1-ml aliquot of the sample was transferred to 0.9 ml of PBS in the second row, and the process was repeated for the remaining rows (to produce five ten-fold dilutions). One microliter from each well was transferred onto standard MacConkey agar for total *E. coli* enumeration and onto MacConkey agar containing 25 μg/ml nalidixic acid, 6.25 μg/ml ampicillin, and 25 μg/ml cephalexin for challenge strain enumeration with a pin-tool replicator. The plates were incubated aerobically (37°C for 24 h). The final dilution of each sample was recorded and entered into the most probable number (**MPN**) calculator for determination of the MPN value of the sample (Berghaus et al., 2013).

#### Antibiotic Susceptibility Testing.

The automated National Antibiotic Resistance Monitoring System (NARMS, Sensititre^®^, USA) was used to determine minimal inhibitory concentrations (**MICs**) for antibiotic resistance levels of 3 random isolates of *E. coli* from antibiotic-free media for each of 240 samples; a total of 720 *E. coli* isolates were used. The MICs of the following antibiotics were tested: ampicillin, amoxicillin-clavulanic acid, azithromycin, cefoxitin, ceftriaxone, chloramphenicol, ciprofloxacin, gentamicin, meropenem, nalidixic acid, streptomycin, sulfisoxazole, tetracycline, and trimethoprim-sulfamethoxazole.

### Statistical Analysis

#### Performance Data.

Statistical evaluations were carried out using Statistical Package for Social Sciences (SPSS 22.0., IBM Corp., US) (SPSS, 2013), and the results were considered significant at *P* < 0.05. After checking the data for normal distribution (Kolmogorov–Smirnov test) and homogeneity of variances (Levene's test), ANOVA, followed by the Bonferroni test, was performed. If variances were not homogenous, the data were evaluated by the Welch test with Tamhane's T2 test as a post hoc test. Data that were not normally distributed were further analyzed by the Kruskal–Wallis test (nonparametric ANOVA), followed by pairwise comparison.

#### E. coli Enumeration.

Analysis was performed using SAS Enterprise software (SAS 9.4 with SAS Enterprise Guide 7.1 © (64bit) 2014 by SAS Institute Inc., Cary, NC, USA).

Linear mixed models were used to compare *E. coli* counts on the basis of MPN results expressed as colony forming unit (**CFUs**) per g of sample between days and treatment groups (MIXED procedure, SAS). Days, treatments and their interactions served as fixed effects and pens and birds as nested random effects. Before starting the statistical analysis, CFUs were log_10_ transformed to obtain linearity (because of the decimal dilution schema used for MPN determination). Using the Tukey–Kramer method, least square means of days as well as treatments were compared at a significance level of 5%.

Additionally, the prevalence of resistant isolates was compared between days and treatment groups using generalized linear mixed models (GLIMMIX procedure, SAS). Logistic regression was applied with the logit function to account for the correlation of isolates obtained from the same pens. Days, treatments, and their interactions served again as fixed effects and pens and birds as nested random effects. Multiple comparisons were applied to test for significant differences of days as well as treatments. Differences between least square means were tested by the Tukey–Kramer method (*α* = 5%). Furthermore, an in-depth analysis of treatment effects on different days was performed to test for specific treatment group differences of interest.

#### Antibiotic Susceptibility Testing.

MICs were log_2_ transformed prior to statistical analysis (based on the MICs, provided in concentration steps of two to the power of n). MICs reported as being greater than the upper limit of the assay or lower than the lower limit of the assay were set as being equal to the corresponding limit to be included in the statistical analysis. Linear mixed models were used again to compare the means of the MICs between days and treatment groups (MIXED procedure, SAS). Model effects were set and statistical analysis was performed in the same manner as described for the MPN results (see “*E. coli* enumeration”).

Additionally, the prevalence of resistant isolates was compared between days and treatment groups using generalized linear mixed models (GLIMMIX procedure, SAS) as described above (“*E. coli* enumeration”). Logistic regression was applied with the logit function for binary response distributions. In the case of multinomial (ordered) response distributions, the cumulative logit function was used. Treatment differences were examined by means of the Tukey–Kramer test.

For multinomial responses, intermediate and resistant prevalence results were merged prior to analysis in order to obtain binary responses. For treatment and day combinations with no intermediate or resistant responses, analysis was conducted without considering the interaction terms in the model. The Tukey–Kramer test was applied again for in-depth analysis of treatment effects on different days to test for specific treatment group differences of interest. Furthermore, contingency analysis was used for interpretation of some of the results regarding resistance.

## RESULTS AND DISCUSSION

### Performance Data

The influence of oral challenge with multi-resistant *E. coli* as well as AB, FA, and SA on poultry performance is shown in Table 1. Oral challenge with APEC did not influence the performance of the birds. The lack of change in performance in the challenged group compared to the non-challenged control shows that the APEC challenge strain did not have a significant impact on bird health and performance, perhaps due to the low competitiveness of APEC with other intestinal microorganisms. Non-significant differences in performance parameters between treatment groups may also be due to the determination of performance parameters per pen, without individual animal data or excessive variability between treatment pens.

**Table 1.** Performance characteristics and standard deviations (± SD) of broilers (400/group) that received ampicillin, a feed additive based on organic acids (FA) or a synbiotic preparation (SA) compared to the control groups.

	NC	CC	AB	FA	SA	*P*-value
Initial weight, g	46 ± 0.62	46 ± 0.89	46 ± 0.24	46 ± 0.51	46 ± 0.37	0.15
BW d17, g	471 ± 18.1	453 ± 15.8	473 ± 50.8	468 ± 16.2	455 ± 29.5	0.32
BW d38, g	1967 ± 102,8	1928 ± 48.8	1972 ± 110.1	1995 ± 63.4	1960 ± 79.3	0.62
ADFI d1–17, g/d	31.0 ± 1.2	30.3 ± 1.0	30.5 ± 2.9	30.8 ± 1.0	30.3 ± 1.7	0.67
ADFI d -38, g/d	149.1 ± 5.1	148.9 ± 5.1	147.9 ± 7.0	146.8 ± 6.3	148.9 ± 7.8	0.95
FCR d1–17, g/g	1.34 ± 0.05	1.37 ± 0.05	1.30 ± 0.05	1.34 ± 0.05	1.36 ± 0.04	0.11
FCR d1–38, g/g	1.64 ± 0.14	1.68 ± 0.06	1.62 ± 0.03	1.62 ± 0.03	1.63 ± 0.04	0.13
Mortality, %	3.00	4.25	3.50	5.75	2.75	0.478

NC, negative control without *E. coli* challenge; CC, *E. coli* challenge control; AB, ampicillin; FA, feed additive based on organic acids; SA, multistrain synbiotic; BW, body weight; ADG, average daily weight gain; ADFI, average daily feed intake; FCR, feed conversion ratio; mean values ± standard errors.

The studies described below present the effects of antibiotics, organic acids, cinnamaldehyde, and synbiotics on growth performance. Penicillins are the most commonly used antibiotics in poultry (Hofacre et al., 2013). Ampicillin is registered for use in poultry in large poultry producing countries such as Brazil, China, Germany, and France (Roth et al., 2018) Stokstad and Jukes (1950), showed that small subtherapeutic doses of penicillin and tetracycline enhance weight gain in poultry. Antibiotics have been used in animals for the treatment of diseases, for the prevention and control of diseases, and as growth promoters (Economou and Gousia, 2015). The administration of antibiotics decreases or alters the bacterial populations present in the digestive tract, which protects animals from pathogenic organisms, increases animal weight and improves meat quality (Fairchild et al., 2001). Antibiotic resistance is the main undesirable side effect of antibiotic use (EFSA/ECDC, European Food Safety Authority and European Centre for Desease Prevention and Control, 2016). Replacement of antibiotics for disease prevention with non-antibiotic substances is essential for implementation of technological solutions that can reduce selection pressure and therefore reduce contamination with AR bacteria.

Organic acids and cinnamaldehyde improve chicken performance via antimicrobial activity (Helander et al., 1998; Raftari et al., 2009; Wang et al., 2009b; Adil et al., 2010). Olarve et al. (2007) showed significant effects on the weight gain and feed efficiency of broilers by using 0.3 and 0.4% (a blend of formic, fumaric, lactic, propionic, and phosphoric acids) in basal diets. The BWs and FCRs of broilers were significantly increased by supplementation with a mixture of formic and propionic acids (Senkoylu et al., 2007). Improvement in weight gain and FCR due to improved nutrient digestibility was detected in a study where formic or fumaric acid and acetic or citric acid were used (Ghazalah et al., 2011). Application of the same synbiotic product as that used in the present study has been previously shown to improve BW gain and FCR as well as the apparent ileal and total tract digestibility (Palamidi et al., 2016). Other studies with the same product showed improvement of zootechnical performance parameters and nutrient digestibility compared to the control (Ritzi et al., 2014; Mountzouris et al., 2015). The tested multistrain synbiotic showed significant modulation of the composition of the cecal microbiota, resulting in increased *Bifidobacterium* spp. and *Lactobacillus* spp. concentrations compared with the control (Mountzouris et al., 2010).

### Microbiological Analysis

#### E. coli From Chick Papers.

Swabs of 20 chick box papers were collected on day 0 and cultured for detection of multi-resistant *E. coli* with resistance to ampicillin, cephalexin, and nalidixic acid. The objective was to confirm the absence of multi-resistant *E. coli* prior to challenge with the multi-resistant APEC strain with resistance to these antibiotics. For the cultures grown on MacConkey agar supplemented with ampicillin, cephalexin, and nalidixic acid, no *E. coli* was identified on any of the 20 swabs. This outcome can be seen as a prerequisite for the planned challenge with the multi-resistant APEC strain.

#### E. coli Enumeration.

*E. coli* counts in cecal samples on MacConkey medium without and with antibiotic supplementation are presented in Table 2, and the statistical evaluation of differences between treatments, days and the interactions between treatments and days is presented in Table 3.

**Table 2.** *E. coli* counts in cecal samples on days 17 and 38 of the trial on MacConkey medium without and with antibiotic supplementation, shown as log_10_ MPN/g values and standard deviations (± SD); 24 positive samples.

Antibiotic	Day	NC	CC	AB	FA	SA
None	17	6.83 ± 1.36	6.78 ± 1.14	7.83 ± 1.01	6.66 ± 1.70	6.27 ± 1.19
	38	6.04 ± 1.35	5.57 ± 1.47	5.23 ± 1.54	4.96 ± 1.2	4.46 ± 1.72
Ampicillin, cephalexin	17	0 (0/24)	0.97 ± 1.21 (16/24)	1.86 ± 0.31 (14/24)	1.66 ± 0.99 (16/24)	1.77 ± 1.18 (14/24)
and nalidixic acid	38	0.68 ± 0.53 (5/24)	1.38 ± 1.24 (11/24)	1.14 ± 1.28 (9/24)	1.46 ± 1.17 (16/24)	1.54 ± 1.38 (15/24)

NC, negative control without *E. coli* challenge; CC, *E. coli* challenge control; AB, ampicillin; FA, feed additive based on organic acids; SA, multistrain synbiotic.

**Table 3.** Statistical evaluation of differences in *E. coli* counts on MacConkey medium without and with antibiotic supplementation among treatments, days, and interactions between treatments and days.

Antibiotic	Effect of day	Effect of treatment	Effect of interaction
None	17>38 (*P* < 0.0001)	NC, AB>SA (*P* = 0.0016) *^,^**	*P* = 0.02
Ampicillin, cephalexin and nalidixic acid	17>38 (*P* = 0.02)	NC<FA, SA (*P* < 0.0001) *	*P* = 0.30
Ampicillin, cephalexin and nalidixic acid	*P* = 0.56)	NC<CC, AB, FA, SA (*P* = 0.0036) ***	n.a.

NC, negative control without *E. coli* challenge; CC, *E. coli* challenge control; AB, ampicillin; FA, feed additive based on organic acids; SA, multistrain synbiotic; n.a., not available.

*MIXED procedure and multiple comparisons of *E. coli* count results adjusted according to the Tukey–Kramer test at a significance level of 5%.

**In-depth analysis of treatment effects by day (Tukey–Kramer test) showed significant differences between AB-SA on day 17 and NC-SA on day 38.

***GLIMMIX procedure with and without interaction terms and in-depth analysis of treatment effects by day showed significant differences between NC-CC, NC-AB, NC-FA, and NC-SA on day 17 only.

The means of the *E. coli* counts in cecal samples grown on MacConkey medium that was not supplemented with any antibiotics were significantly high on day 17. Significant effects of treatment were observed between the NC-SA and AB-SA groups on both days. As the interaction term was also significant, in-depth analysis (multiple comparisons, Tukey–Kramer) showed significant differences between treatments AB-SA on day 17 and NC-SA on day 38, with low *E. coli* counts in SA observed in both cases. The influence of synbiotics on *E. coli* counts has also been shown in other studies. Gunal et al. (2006) demonstrated that probiotic supplementation decreased the abundances of gram-negative bacteria compared to the control group. The population of intestinal *E. coli* in broilers that were fed lactobacilli-supplemented feed was significantly lower (*P* < 0.05) than that of the control (Jin et al., 1996).

For the analysis of *E. coli* counts in cecal samples grown on MacConkey medium containing antibiotic supplements, all samples that showed a lack of growth were excluded from the statistical analysis. No *E. coli* growth was observed on day 17 in the NC group. Because a zero value would be undefined on the log scale after log_10_ transformation, this group could not be considered for comparison of means. Mixed model analysis indicated that the effects of the days and treatments were not significant. To include all the results, the detection limit (MPN code 0–0-0) was set to 0.3 CFU. This value was based on the fact that the MPN assay results, with MPN code 0–0-0, corresponded to a value of < 0.3 CFU per ml of medium. Under these conditions, we observed significant differences among treatments as well as days, whereas the interactions between the two had no significant effects, as per the linear mixed model analysis. Consequently, significant differences between the NC-FA and NC-SA groups could be identified using the Tukey–Kramer test for multiple comparisons.

Furthermore, based on the growth ability in the presence of antibiotic substances, the prevalence of multi-resistant isolates was compared between days and treatment groups using the *E. coli* count results in cecal samples on MacConkey medium supplemented with ampicillin, cephalexin, and nalidixic acid. When considering all culture-positive samples that exhibited growth on MacConkey agar as resistant and all other results as susceptible to the applied antibiotic mixture, categorical data analysis showed no day-related effects but significant treatment-related effects. No multi-resistant *E. coli* strain with resistance to ampicillin, cephalexin and nalidixic acid was detected in NC on day 17 of the trial, but a strain was detected on day 38 of the trial, indicating transition of resistance determinants between pens, despite separation with two empty pens between groups. Because there was no resistant strain in group NC on day 17, the interaction term had to be excluded for successful application of the GLIMMIX procedure. In-depth analysis showed significant differences between the NC-CC, NC-AB, NC-FA, and NC-SA groups on day 17 only. The *E. coli* count results and prevalence of *E. coli* are summarized by sampling day and treatment group in Table 2 and statistical evaluation in Table 3. *E. coli* were detected in all tested cecal samples except the NC group on day 17. No multi-resistant *E. coli* that were resistant to ampicillin, cephalexin, and nalidixic acid were detected in the negative control group on day 17 of the trial. However, resistant *E. coli* was detected in 21% (5/24) of samples in the negative control on day 38 of the trial, indicating the possible transmission of the multi-resistant APEC strain used for the oral challenge to the negative control.

#### Antimicrobial Susceptibility Testing.

The mean MIC results and the corresponding standard deviations expressed as log_2_ values are shown in Table 4, and the statistical evaluation is presented in Table 5.

**Table 4.** Mean minimal inhibitory concentrations (MICs) of tested antibiotics and the corresponding standard deviations (± SD), shown as log2 values.

	Antibiotic	NC	CC	AB	FA	SA
Day 17	n	72	72	72	72	71
	Amoxicillin—clavulanic acid	1.38 ± 1.11	1.75 ± 1.2	3.24 ± 1.31	1.40 ± 0.80	1.08 ± 0.69
	Ampicillin	1.58 ± 1.55	1.71 ± 1.58	4.33 ± 1.39	1.44 ± 1.09	0.90 ± 0.74
	Azithromycin	2.60 ± 1.08	2.17 ± 0.67	1.79 ± 0.63	2.33 ± 0.92	2.15 ± 0.75
	Cefoxitin	2.36 ± 0.91	2.43 ± 0.90	3.21 ± 1.30	2.51 ± 0.69	2.06 ± 0.67
	Ceftriaxone	−1.76 ± 1.14	−1.79 ± 1.01	0.22 ± 2.71	−2.00 ± 0.00	2.00 ± 0.00
	Chloramphenicol	2.53 ± 0.67	2.49 ± 0.56	2.32 ± 0.53	2.64 ± 0.56	2.56 ± 0.67
	Ciprofloxacin	−5.91 ± 0.62	−5.89 ± 0.63	6.06 ± 0.00	−5.93 ± 0.33	6.06 ± 0.00
	Gentamycin	2.04 ± 2.34	1.79 ± 2.27	2.18 ± 2.34	2.33 ± 2.32	2.06 ± 2.15
	Meropenem	−4.06 ± 0.00	−4.06 ± 0.00	4.06 ± 0.00	−4.06 ± 0.00	4.06 ± 0.00
	Nalidixic acid	1.15 ± 1.02	1.15 ± 1.02	0.74 ± 0.56	1.22 ± 0.56	0.85 ± 0.40
	Streptomycin	4.57 ± 1.67	4.47 ± 1.54	4.60 ± 1.38	4.61 ± 1.67	4.96 ± 1.26
	Sulfisoxazole	6.51 ± 1.94	5.35 ± 1.80	5.49 ± 1.87	5.72 ± 1.92	6.04 ± 2.00
	Tetracycline	4.42 ± 1.20	4.04 ± 1.41	3.04 ± 1.44	4.25 ± 1.31	4.54 ± 1.09
	Trimethoprim-sulfamethoxazole	−2.02 ± 1.79	−2.59 ± 1.24	1.89 ± 2.10	−2.75 ± 1.17	2.63 ± 1.42
Day 38	n	72	72	72	72	72
	Amoxicillin—clavulanic acid	2.18 ± 09.94	2.49 ± 1.07	3.51 ± 1.06	2.14 ± 0.83	2.18 ± 0.84
	Ampicillin	2.24 ± 1.51	2.93 ± 1.76	4.96 ± 0.35	2.29 ± 1.42	2.51 ± 1.59
	Azithromycin	2.92 ± 0.98	2.36 ± 0.59	2.31 ± 0.66	2.68 ± 0.82	2.63 ± 0.57
	Cefoxitin	2.69 ± 0.74	2.79 ± 0.87	3.36 ± 1.18	2.78 ± 0.56	2.53 ± 0.63
	Ceftriaxone	−1.76 ± 1.14	−1.53 ± 1.58	0.29 ± 2.67	−1.92 ± 0.71	2.00 ± 0.00
	Chloramphenicol	2.81 ± 0.46	2.76 ± 0.46	2.67 ± 0.50	2.93 ± 0.42	2.90 ± 0.48
	Ciprofloxacin	−5.73 ± 0.90	−5.88 ± 0.66	6.02 ± 0.35	−5.81 ± 0.67	5.98 ± 0.50
	Gentamycin	3.61 ± 1.24	2.72 ± 1.99	3,08 ± 1.81	2.93 ± 1.95	2.67 ± 1.92
	Meropenem	−4.06 ± 0.00	−4.06 ± 0.00	4.06 ± 0.00	−4.06 ± 0.00	4.06 ± 0.00
	Nalidixic acid	1.61 ± 1.19	1.44 ± 0.98	1.08 ± 0.64	1.56 ± 0.89	1.18 ± 0.70
	Streptomycin	5.71 ± 0.72	5.18 ± 1.17	5.28 ± 1.05	5.21 ± 1.22	5.51 ± 0.93
	Sulfisoxazole	7.57 ± 1.23	6.39 ± 1.89	6.40 ± 1.96	6.56 ± 1.85	6.78 ± 1.86
	Tetracycline	4.83 ± 0.69	4.79 ± 0.77	4.13 ± 1.37	4.58 ± 1.04	4.71 ± 0.90
	Trimethoprim-sulfamethoxazole	−1.67 ± 1.97	−2.03 ± 1.76	0.60 ± 2.49	−2.42 ± 1.43	1.99 ± 1.97

Three isolates were evaluated from each of 3 birds per pen in each of 8 pens per treatment group (72 isolates per treatment group); mean ± standard error; NC, negative control without *E. coli* challenge; CC, *E. coli* challenge control; AB, ampicillin; FA, feed additive based on organic acids; SA, multistrain synbiotic.

**Table 5.** Statistical evaluation of MICs and standard deviations (± SDs) of the antibiotics showing significant differences among treatments, days, and interactions between treatments and days.

Antibiotic	Effect of day	Effect of treatment*	Effect of interaction
Amoxicillin—clavulanic acid	38>17 (*P* < 0.0001)	AB>NC, CC, FA, SA (*P* < 0.0001)**	*P* < 0.0001
Ampicillin	38>17 (*P* < 0.0001)	AB>NC, CC, FA, SA (*P* < 0.0001)	*P* < 0.0001
Azithromycin	38>17 (*P* < 0.0001)	NC>AB (*P* = 0.02)	*P* = 0.11
Cefoxitin	38>17 (*P* < 0.0001)	AB>SA (*P* = 0.02)	*P* = 0.24
Ceftriaxone	*P* = 0.38	AB>FA, SA (*P* = 0.03)	*P* = 0.44
Chloramphenicol	38>17 (*P* < 0.0001)	*P* = 0.14	*P* = 0.93
Ciprofloxacin	38>17 (*P* = 0.04)	*P* = 0.25	*P* = 0.44
Gentamicin	38>17 (*P* < 0.0001)	*P* = 0.81	*P* = 0.03
Nalidixic acid	38>17 (*P* < 0.0001)	*P* = 0.05	*P* = 0.86
Streptomycin	38>17 (*P* < 0.0001)	*P* = 0.72	*P* = 0.09
Sulfisoxazole	38>17 (*P* < 0.0001)	*P* = 0.14	*P* = 0.85
Tetracycline	38>17 (*P* < 0.0001)	AB<NC, SA (*P* = 0.02)***	*P* < 0.0001
Trimethoprim-sulfamethoxazole	38>17 (*P* < 0.0001)	*P* = 0.17	*P* < 0.002

NC, negative control without *E. coli* challenge; CC, *E. coli* challenge control; AB, ampicillin; FA, feed additive based on organic acids; SA, multistrain synbiotic.

*MIXED procedure and multiple comparisons of the MICs adjusted according to the Tukey–Kramer test at a significance level of 5%.

**In-depth analysis did not show significant differences between CC-AB on day 38.

***In-depth analysis did not show significant differences between NC-AB, AB-FA, and AB-SA on day 17 only.

Generally, the mean MICs on day 38 were higher for all antibiotic substances compared to those on day 17, with the exception of the MICs of ceftriaxone. These results indicated higher antibiotic resistance levels on day 38 compared to day 17. Antibiotics were not used between days 16 and 38; therefore, the increase in MIC and resistance to antibiotics may not be due to selective pressure. However, resistant *E. coli* can compete with susceptible strains in the absence of selective antibiotic pressure (Smith et al., 2007). The incorporation and development of resistant *E. coli* strains in the intestinal tract depends on the composition of the intestinal microbiota, growth rates, transmission dynamics, persistence, and features affecting colonization, such as adherence and virulence (Karami et al., 2006; Marciano et al., 2007). All of these factors together affect the epidemiological fitness of resistant *E. coli* and the ability of these bacteria to competitively develop in the intestinal environment (Sundqvist, 2014).

Significant effects of treatment were evident with amoxicillin-clavulanic acid, ampicillin, azithromycin, cefoxitin, ceftriaxone, and tetracycline, whereas the AB group often exhibited a different behavior. The MICs of amoxicillin-clavulanic acid and ampicillin exhibited significant treatment-related effects as well as effect of the interactions between days and treatments. Further analysis confirmed that the ampicillin-treated AB group exhibited a greater mean MIC than any other group. Additionally, the significance of the interaction term was evident with regard to tetracycline, trimethoprim-sulfamethoxazole, and gentamycin, whereas significant differences between treatments could be verified only in the case of tetracycline.

The effect of selective pressure of ampicillins on the increased MIC values of penicillins is clearly recognizable here. However, the selective pressure of ampicillin reduced the MIC values of tetracyclines with the *E. coli* isolates. In the present study, the MIC value of tetracycline was significantly lower in the AB group than in NC and SA. Antibiotic use may also lead to decreased abundances of some AR bacteria. The ability of enrofloxacin to decrease the prevalence of extended-spectrum beta-lactamase-producing *E. coli* was demonstrated by Roth et al. (2017). The effect of the applied FA and synbiotic preparation on the prevalence of resistant *E. coli* could be seen in the distribution of MIC values for cefoxitin- and ceftriaxone-resistant *E. coli*. The MIC of cefoxitin was lower in the SA group, and the MIC of ceftriaxone was lower in the FA and SA groups, than in the AB group.

Given the antibiotic breakpoints defined by the Clinical and Laboratory Standards Institute (2012), MICs can be classified as resistant, susceptible or intermediate. Because there are no CLSI data for interpretation of azithromycin, nalidixic acid, and streptomycin MICs, the prevalence of resistant isolates could not be investigated. Based on the prevalence classification of the MIC results (see Table 6), the prevalence of some resistant isolates (sulfisoxazole, tetracycline, trimethoprim-sulfamethoxazole, and gentamycin) could be successfully analyzed with the GLIMMIX procedure of SAS. For these antibiotic substances, again, the day-related effects were significantly higher for resistant isolates on day 38, whereas, with the exception of the effects observed with tetracycline, no treatment-related or interaction-related effects could be identified. For tetracycline, some significance was observed between the prevalence results of AB-SA on day 17, in which the AB group exhibited the lowest abundance of resistant isolates.

**Table 6.** Statistical evaluation of the resistance to antibiotics, showing significant differences among treatments, days, and interactions between treatments and days.

Antibiotic	Effect of day	Effect of treatment	Effect of interactions
Amoxicillin—clavulanic acid***	38>17 (*P* = 0.036)	AB>NC, CC, FA, SA (*P* < 0.0001)	n.a.
Ampicillin**	38>17 (*P* < 0.0001)	AB>NC, CC, FA, SA (*P* < 0.0001)	n.a.
Cefoxitin***	*P* = 0.36	AB>NC, CC, FA, SA; SA<CC(*P* < 0.0001)	n.a.
Ceftriaxone***	*P* = 0.69	AB>NC, CC, FA, SA (*P* < 0.0001)	n.a.
Chloramphenicol**	*P* = 0.65	*P* = 0.49	n.a.
Gentamicin*	38>17 (*P* < 0.0001)	*P* = 0.70	*P* = 0.17
Sulfisoxazole*	38>17 (*P* < 0.0001)	*P* = 0.11	*P* = 0.75
Tetracycline*	38>17 (*P* < 0.0001)	*P* = 0.04****	*P* = 0.30
Trimethoprim-sulfamethoxazole*	38>17 (*P* = 0.0002)	*P* = 0.16	*P* = 0.48

NC, negative control without *E. coli* challenge; CC, *E. coli* challenge control; AB, ampicillin; FA, feed additive based on organic acids; SA, multistrain synbiotic; n.a., not available.

*GLIMMIX procedure and multiple comparisons of the resistance results adjusted according to the Tukey–Kramer test at a significance level of 5%.

**GLIMMIX procedure without interaction terms and in-depth analysis of treatment effects by day according to the Tukey–Kramer test.

***Interpretation by contingency analysis (dependencies in contingency tables of treatments were tested by Pearson's Chi-squared test, and tests on subgroups were based on the Bonferroni correction to comply with the type I error rate).

****In-depth analysis showed significant differences between AB-SA on day 17 only.

Due to the unbalanced response matrix of some of the antibiotic substances (amoxicillin-clavulanic acid, ampicillin, cefoxitin, ceftriaxone, and chloramphenicol), the convergence criteria of the model (GLIMMIX procedure, SAS) could not be met. Therefore, the interaction term was excluded from the model in order to allow statistical analysis. Neither day- nor treatment-related effects were observed for chloramphenicol-resistant *E. coli*. Higher abundances (*P* < 0.05) of ampicillin-resistant were observed on day 38 than on day 17. There were significant differences between treatments, with the highest rate of ampicillin resistance observed in the AB group. The increased abundance of AR bacteria due to oral administration of antibiotics in the AB group corresponds with the outcome of the literature review for poultry conducted by Simoneit et al. (2015).

In the remaining 3 cases (amoxicillin-clavulanic acid, cefoxitin and ceftriaxone), analysis and interpretation of the resistance results was based on contingency analysis. Significant differences in the distribution of amoxicillin-clavulanic acid-resistant isolates were detected with respect to days and treatments, while for the cefoxitin- and ceftriaxone-resistant isolates, significant differences were observed with respect to only treatments. All 3 cases showed similarities in response patterns with the most resistant isolates in the AB group and, interestingly, with almost no resistant isolate on day 17 in groups FA and SA. Supplementation of the diet with FA in another trial contributed to a significant decrease (*P* < 0.05) in the abundance of *E. coli* that was resistant to ampicillin and tetracycline compared to the control and enrofloxacin-supplemented groups (Roth et al., 2017).

For azithromycin, ciprofloxacin, meropenem, nalidixic acid, and streptomycin, no statistical analysis could be performed because there was no variation in the resistance results (either the bacteria were susceptible or the results were not interpretable).

## CONCLUSION

A high prevalence of AR *E. coli* in all experimental groups was observed throughout the study. It may be concluded that administration of ampicillin for 5 d led to a significant increase in the abundances of *E. coli* resistant to ampicillin, amoxicillin-clavulanic acid, cefoxitin, and ceftriaxone, all of which are ß-lactam antibiotics. The tested feed additives did not increase the prevalence of resistant determinants in the guts of the broilers. Moreover, the MIC of ceftriaxone was lower in the FA and SA groups than in the AB group. Additionally, administration of SA led to a decreased MIC value of cefoxitin in the SA group. Further studies are needed to confirm these findings and to clarify the mode of action of FA and SA on *E. coli* strains resistant to cephalosporin and ß-lactams in the digestive tract.
